# The difficulties of reproducing conventionally derived results through 500k-chip technology

**DOI:** 10.1186/1753-6561-3-s7-s66

**Published:** 2009-12-15

**Authors:** Hans H Stassen, Katrin Hoffmann, Christian Scharfetter

**Affiliations:** 1 Psychiatric University Hospital, PO Box 1931, CH-8032 Zurich, Switzerland; 2 Institute of Medical Genetics, Charité, Humboldt University, D-13353 Berlin, Germany

## Abstract

Based on a "training" sample of 1,042 subjects genotyped for 5,728 single-nucleotide polymorphisms (SNPs) of a conventional 0.4-Mb genome scan and a "test" sample of 746 subjects genotyped for 545,080 SNPs on a 500k-chip, we investigated the extent to which the subjects' immunoglobulin M levels can be reproducibly predicted from a multilocus genotype. We were specifically interested in the reproducibility of predictors across populations (1,042 versus 746 subjects) and across SNP sets (conventional genome scan versus anonymous 500k-chip) because this is a prerequisite for clinical application. For the training sample, neural network (NN) analysis yielded classifiers that predicted immunoglobulin M levels from the subjects' multilocus genotypes at acceptable error rates through a configuration of 15 genomic loci (61 SNPs). With the test sample (746 subjects) we addressed the question of reproducibility across populations and across SNP sets by means of a novel "competitive SNP set" approach. However, the chip data contained several sources of distortion, including greatly elevated noise levels and artifact-prone SNP regions, thus complicating attempts to verify the reproducibility of NN predictors. Though 5 of 15 genomic loci from the training samples appeared to be reproducible, the NN classifiers derived so far from the test samples are insufficiently compatible with the training samples. Nonetheless, our results are promising enough to justify further investigations. Because the underlying algorithm can easily be split into parallel tasks, the proposed "competitive SNP set" approach has turned out to be well suited for computers with today's 64-bit multiprocessor architectures and to offer a valuable extension to genome-wide association analyses.

## Background

In this investigation we focused on the immunoglobulin M (IgM) phenotype because a heritable malfunction of the inflammatory response system has been linked to various complex diseases. The "natural" antibody IgM displays a high within-pair concordance in monozygotic twins in the range of 0.849 ± 0.091, while chronically elevated IgM levels develop years before the first clinical symptoms occur [1]. Thus, chronically elevated IgM levels have been hypothesized to be related to a heritable malfunction in the inflammatory response system. To investigate the extent to which IgM levels can be reproducibly predicted for each individual patient from his/her multilocus genotype, we aimed at carrying out a neural network (NN) analysis with a sufficiently large sample (Genetic Analysis Workshop (GAW) 15: n = 1,042 subjects genotyped for 5,728 SNPs of a 0.4-Mb genome scan) under the constraint of a 10-fold cross-validation. Because NN results tend to be over-optimistic, we were specifically interested in the reproducibility of predictors across populations ("training" versus "test" samples) and across single-nucleotide polymorphism (SNP) sets (conventionally designed genome scan versus anonymous 500k-chip). To address these issues, we used independent test samples (GAW16: n = 746 subjects genotyped for 545,080 SNPs of a 500k-chip) along with six different SNP sets, each with 5,728 SNPs drawn from the 500k-chip under the constraint of maximum informativeness and compatibility with the training SNPs.

## Methods

### NN analysis

**Figure 1:**
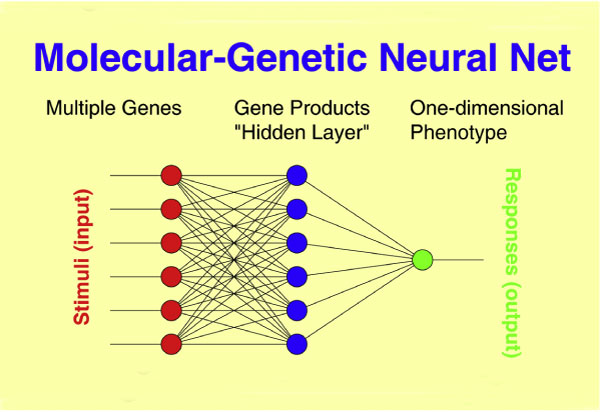
**Molecular-genetic neural network analysis**. Molecular-genetic NN that connects multiple genetic factors as observed in each individual patient through a layer of gene products to a one-dimensional phenotype, for example, the IgM level.

**Figure 2:**
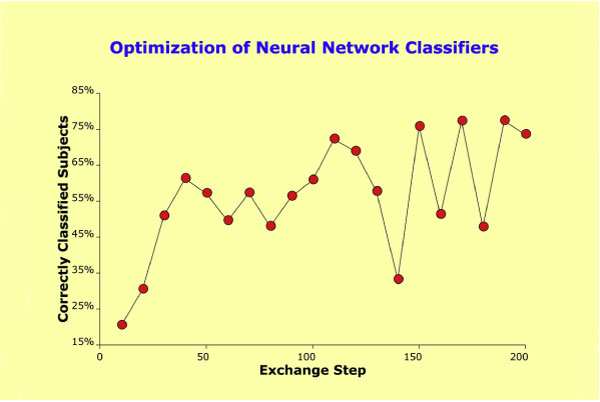
**Optimization of neural network classifiers**. Iterative optimization of the starting configuration by systematically adding/removing genomic loci while fitting the NN model to the set of 1,042 observations under the constraint of reproducibility with ten-fold cross-validation. The red circles designate the percentage of correctly classified subjects for each optimization step, with optimization steps being plotted along the x-axis (over-proportionally large drops in performance indicate removal of loci of larger weight).

Standard (logistic) regression connects genotype with phenotype in a direct way, thus greatly simplifying biology. In fact, genes code for proteins or RNA ("gene products"), which may interact in a variety of ways and influence the phenotype only after a cascade of intermediate steps. Molecular-genetic NNs generalize standard regression analysis in a very natural way by 1) implementing multistage gene products through one or more intermediate "layer(s)" (Fig. 1), and 2) allowing for (linear/nonlinear) interactions between genes and between gene products. It is the advantage of NNs that the knowledge about the cascade of intermediate steps, which ultimately lead from genotype to phenotype, can be incomplete or even unknown ("hidden layers"). In this case, the model's gene product layers lack direct interpretation and act in the manner of a "black box" [2]. However, the influence of each single gene on the phenotype, as well as the interactions between genes, can be quantified and detailed through analysis of the weight matrices of the fitted model.

The most popular learning strategy, the back-propagation algorithm, looks for the minimum of the error function in the weight space (goodness of fit) using the method of gradient descent. The basic algorithm is:

(i) output:      *y*_i _observed   (*i *= 1,2,...*N*_i_)

(j) hidden layer:         (*j *= 1,2,...*N*_j_)

(k) input:   *s*_*k *_= *x*_*k*_   *x*_*k*_observed   (*k *= 1,2,...*N*_k_)

   improvements:      (ν = 1,2,..*p*)

where *x*_*k *_denote observed genotypes, *y*_*i *_observed phenotypes, *σ *the activation function of sigmoid-type: R → (0,1), *α *the learning rate, and *p *the number of subjects. This algorithm can easily be adapted to the requirements of specific genetic analyses.

### *k*-Fold cross-validation

Results derived through the standard NN approach, which uses 80% of samples for training and the remaining 20% for testing, tend to be over-optimistic, in particular if genotype errors and missing data are present. Therefore, in the *k*-fold cross-validation, the data are split into *k *roughly equal parts, and *k*-1 partitions are used for training, while one partition is used for testing. This process is repeated until each partition has served as a test set, so that *k *estimates of prediction error are generated. The resulting prediction error is approximately unbiased for the "true" error if *k *is sufficiently large (*k *≈ 10 is a typical value in practice).

### Study population

Our training sample comprised 511 nuclear and multiplex families with 1,073 subjects ascertained through index cases with a diagnosis of rheumatoid arthritis. All subjects were genotyped for 5,728 specifically selected SNPs of a conventionally designed genome scan resolving an average 0.4-Mb inter-marker distance (GAW15) [3]. As intra-lab (9-15%) and inter-lab (10-20%) variations of IgM measures do not allow NN analyses of raw data, we defined three IgM categories that appeared to be suitable for clinical use: 1) normal: 0 ≤ IgM < 13.5; 2); low: 13.5 ≤ IgM < 50; and 3) elevated: 50 ≤ IgM. Due to incomplete data 31 subjects had to be excluded from the analysis, so our training sample included a total of 1,042 subjects. Our replication sample comprised those 746 subjects of the GAW16 rheumatoid arthritis study for whom IgM levels and 500k-chip data were available.

### Two-stage optimization procedure

In a first step ("screening") we looked for clusters of at least three SNPs within a 0.5-Mb region that contributed to the observed IgM distribution in terms of NN performance rates above a prespecified threshold under the constraint of a ten-fold cross-validation. The threshold was chosen in such a way that less than 10% of the SNPs under investigation entered the subsequent optimization phase that relied on the resulting clusters as SNP "pool". In the second step, we constructed NN classifiers in a systematic way by iteratively adding or removing genomic loci from the SNP "pool" and fitting the NN model to the 1,042 observations under the constraint of a ten-fold cross-validation. The number of correctly classified subjects, averaged across the ten solutions, served as an optimization criterion. Our NN approach included a single intermediate layer with the number of gene products equalling the number of genomic loci, while a single output node formed the one-dimensional phenotype (Figure 1).

## Results

### Training samples: predicting IgM level from genotype

The initial screening step revealed 80 clusters of at least three SNPs within 0.5-Mb regions that then served as the SNP "pool" for the construction of NN classifiers. Averaged across the ten solutions and applied to the 1,042 probes, weight matrices and classifiers yielded an (estimated) overall performance for each optimization step. The optimization stopped when a plateau was reached at a rate of 77.3% (± 0.636) correctly classified subjects (Figure 2), a rate that was within the expected range based on the data from monozygotic twins. The final configuration included 15 genomic loci (61 SNPs) that later on served as reference in the replication analyses.

### Compatibility between training and test samples

Of the initial 5,728 training SNPs, only 2,179 (38.0%) were included in the 500k-chip used with the test samples. In most cases, however, at least 8 SNPs were found in the ± 0.1 Mb surrounding interval, except for 121 genomic regions (2.1%) insufficiently covered by the chip. As for informativeness, 33.4% of the chip SNPs displayed insufficient allelic variation, essentially raising the overall "noise" level. By contrast, this latter rate was as low as 4.4% for the training set. IgM prevalences showed significant differences between training sample (62.1% normal, 24.7% low, and 13.2% elevated) and test sample (52.3% normal, 29.6% low, and 18.1% elevated) by design. These differences, however, were irrelevant for the envisaged replication analyses.

### "Competitive SNP set" approach: selecting SNP sets from 500k-ship

**Figure 3:**
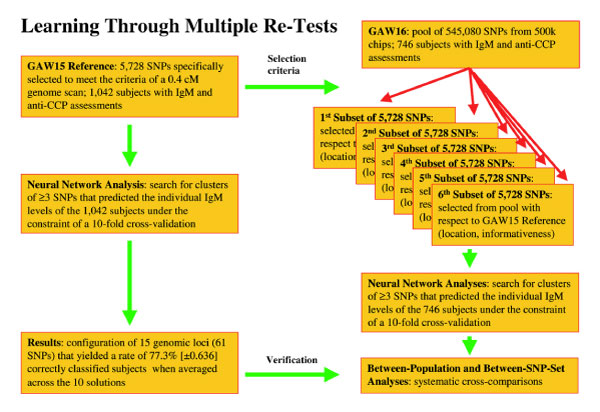
**Learning through multiple re-tests**. Based on independent training samples (n = 1,042; genotyped for 5,728 SNPs of a conventional 0.4-Mb genome scan) and test samples (n = 746; genotyped for 545,080 SNPs of a 500k-chip), we investigated the extent to which the subjects' individual IgM levels can be reproducibly predicted from a multilocus genotype. To test the reproducibility of predictors across populations and across SNP sets, a "competitive SNP set" approach was used to extract six subsets of 5,728 SNPs each from the 500k-chip data according to the criteria: 1) close vicinity to the original training set; 2) maximum informativeness; and 3) <20% overlap between the six subsets.

Based on NCBI36 data, the coordinates X^k ^of the 5,728 SNPs of our training sample were used to define surrounding X^k ^± 0.1 Mb intervals (k = 1,2,...,5,728). Typically 50 to 80 SNPs of the 500k-chip were located in these intervals and served as pool for selecting eight "optimal" SNPs in terms of informativeness and vicinity to the original loci at X^k ^(k = 1,2,...,5,728). Finally, six subsets of 5,728 SNPs each were constructed by randomly combining SNPs from each interval (Figure 3). This process led to mutual overlaps between the six subsets in the range of 14.6 to 16.6%. Due to missing data, typically 40 SNPs (0.7%) of the resulting sets had to be excluded from analysis.

### Test samples: reproducibility of NN predictors

When applied separately to the six competitive SNP sets, the screening procedure of our two-stage optimization algorithm yielded 55 to 74 clusters of at least three SNPs within 0.5-Mb regions. This is a somewhat smaller yield compared to that of the training sample, yet a presumable consequence of the lower allelic variability found in the chip data. Subsequent optimization steps yielded relatively reproducible results (across four out of six SNP sets) for 5 of the 15 genomic loci derived from the training samples (APOB [chromosome 2], CHMP2B [chromosome 3], TFNa [chromosome 6], TBP [chromosome 6], and NLRP7 [chromosome 19]), whereas the most stable regions for the test samples appeared to be mere artifacts inherent in the 500 k chip: 1) across all six SNP sets (13: *18407432*-*18467428*), (20: *22347*-*24962*), and 2) stable across five out of six SNP sets (3: *127908352*-*127934624*), (7: *64334980*-*64399828*), (10: *42184144*-*42240840*), (12: *132000536*-*132033000*), (17: *524930*-*565912*), (19: *18062756*-*18176628*). In consequence, the NN classifiers so far derived from the test samples showed insufficient compatibility with those of the training samples and still require improvements.

## Discussion

Given the strong evidence for the involvement of inflammatory processes in the pathogenesis of various complex illnesses, attempts to "explain" chronically elevated IgM levels in the individual patient through genotype patterns is quite intriguing, as this would offer the opportunity for early intervention before the onset of clinical manifestations. While a conventionally designed 0.4-Mb genome scan enabled the construction of multilocus classifiers through NN analysis, genome-wide association approaches on the basis of 500 k chip data have turned out to involve several sources of distortion, including greatly elevated noise levels and artifact-prone SNP regions. These difficulties have complicated our attempt to verify the reproducibility of NN predictors across populations and across SNP sets - a prerequisite for clinical application - through a novel "competitive SNP set" approach. Nonetheless, our results so far are promising enough to justify further investigation.

## Conclusion

Molecular-genetic NN analyses provide powerful tools when analyzing complex genetic processes where multistage gene products and (linear/nonlinear) interactions between genes and between gene products play a central role (as is the case with most biological phenomena). While the ultimate goal of molecular-genetic research is the detection of "causality", clinicians are also interested in reliable classification and prediction through "objective" laboratory methods. Because the underlying algorithm can easily be split into parallel tasks, the proposed "competitive SNP set" approach is well suited for computers with today's 64-bit multiprocessor architectures, and thus a valuable extension of standard genome-wide association analyses.

## List of abbreviations used

GAW: Genetic Analysis Workshop; IgM: Immunoglobulin M; NN: Neural network; SNP: Single-nucleotide polymorphism.

## Competing interests

The authors declare that they have no competing interests.

## Authors' contributions

All three authors contributed equally to the design of this project and its practical realization; HHS conducted the CPU-intensive computations on a large-scale computer system.
